# The impact of an integrated electronic immunization registry and logistics management information system (EIR-eLMIS) on vaccine availability in three regions in Tanzania: A pre-post and time-series analysis

**DOI:** 10.1016/j.vaccine.2019.10.059

**Published:** 2020-01-16

**Authors:** Sarah Skye Gilbert, Ngwegwe Bulula, Emmanuel Yohana, Jenny Thompson, Emily Beylerian, Laurie Werner, Jessica C. Shearer

**Affiliations:** a PATH, PO Box 900922, Seattle, WA 98109, United States; b Ministry of Health, Community Development, Gender, Elderly and Children, Government of Tanzania, University of Dodoma, Faculty of Social Science in Community Development, Building No. 11, P.O.BOX 743, 40478 Dodoma, Tanzania

**Keywords:** Digital health, Supply chain, Information system, Immunization, Tanzania, Stock notification

## Abstract

**Background:**

Since 2016, the Government of Tanzania has been implementing TImR, an integrated Electronic Immunization registry-logistics management information system (EIR-LMIS) that includes stock notifications. The objective of this study is to estimate the impact of this intervention on vaccine availability.

**Methods:**

Monthly stock-out data were collected from paper registers at facilities, an Excel-based system at districts, and the new system (TImR) across all 924 health facilities in Arusha, Tanga and Kilimanjaro Regions. Six months of stockout rates pre- and post-introduction, by antigen, were compared via a two-way analysis of variance (ANOVA). A mixed-effects logistic regression model with the TImR data identified predictors of vaccine availability across antigens.

**Findings:**

Post-introduction, ANOVA models estimated that overall stock-out rates declined from a monthly average of 7.1% to 2.1% *(p* < 0.01). Three specific vaccines had fewer stock-outs; OPV’s monthly average dropped from 12.5% to 2.1% (*p* < 0.01), MR from 9.4% to 1.0% (*p* < 0.01) and DTP-HepB-HiB from 8.1% to 1.7% (*p* < 0.01). In the mixed-effects logistic regression model, controlling for antigen, odds of stock-out were 4.1% (95% CI: 3.3 – 4.9) lower for each week of tenure. Compared to DTP-HepB-HiB vaccine, odds of BCG vaccine being stocked out were 4.31 as high (95% CI: 3.1 – 5.0). The odds of being stocked-out were 29.7% lower for PCV (95% CI: 8.8 – 45.8) and 26.6% (95% CI: 3.4 – 44.1) lower for rotavirus vaccines compared to DTP-HepB-HiB. The odds of stock out were 37.7% lower for MR vaccine than DTP-HepB-HiB (95% CI: 18.1 – 52.6).

**Conclusions:**

Tanzania’s integrated EIR-eLMIS may increase vaccine availability compared to its paper and Excel based system. Post-introduction of an eLMIS, the odds of a vaccine stock-out reduced over time. Further research could determine the impact of this intervention on vaccine wastage and replenishment response times.

## Highlights

• Digital supply chain information system linked to immunization registry, serves 6M.
• Digital system includes stock notifications, dashboards and peer networks.
• Overall stockout rate significantly lower with digital system vs. legacy system.
• More time with digital system increases odds of vaccine availability.

## 1 Introduction

### 1.1 Digital information systems to strengthen vaccine supply chains

To successfully reach children with life-saving vaccines, vaccine supply chains must achieve the six “rights”, as identified by WHO: right product, right quantity, right condition, right place, right time and right cost [1], [2]. Achieving each of those ‘rights’ requires the use of information. For example, getting the right product in the right quantity at the right place and time requires information about the size of the target population for that product. Traditionally, logistics management information systems (LMIS) have been the tool used by EPI programs to collect and manage supply chain information. The shift towards digital systems – electronic LMIS (eLMIS) – has increased the potential to improve the accuracy and efficiency of supply chain management and the overall supply chain by increasing availability, reducing wastage, and for products like vaccines, monitoring the conditions of the cold chain to ensure products remain potent [1], [3], [4], [5].

Unfortunately, many LMISs today remain predominantly paper-based with incomplete data, poor data quality, large lag times between data entry and availability for use, and lack of use [3], [4], [6], [7]. Even when adopted, eLMIS’s struggle with poor network connectivity, lack of electricity, and change management among users with low digital literacy [8], [9], [10], [11], [12], [13]. Finally, in low-resource contexts, eLMIS demand forecasting is rarely integrated at the facility level with Electronic Immunization Registries (EIRs) where those individual-level routine systems exist, with a notable exception in Vietnam [14]. Whereas eLMIS-only systems generally rely on aggregate, historical data to understand demand, a combined EIR-eLMIS can forecast future vaccine demand based on the schedules of individual children, and improve accuracy of the eLMIS by reducing duplicative data entry and tying each consumed dose to an individual child’s immunization status. Unfortunately, sparse evidence from immunization programs in low-resource settings exists to validate the value of EIR-eLMIS integration. The recently-published WHO guideline revealed that very few experimental or quasi-experimental studies estimate the impact of eLMIS interventions [15], [16]. To address this evidence gap, this study aims to estimate the impact of an integrated EIR-eLMIS intervention, which includes stock notifications, on vaccine availability.

### 1.2 Tanzania immunization program

Tanzania’s Expanded Program for Immunization (EPI) program provides free immunizations to Tanzania’s full birth cohort of 2.184M children. By 2016, the program expanded from the original five vaccines to nine vaccines protecting against 12 antigens, including an HPV vaccine demonstration project in Kilimanjaro Region [17]^1^. Its program is very effective in reaching children, with 2018 WUENIC estimates for third dose of DTP-containing vaccine (DTPcV) at 98% [17].

Prior to the launch of Tanzania’s EIR-eLMIS in 2016, its immunization information system was digital down to the district level, and paper-based at health facility level. District managers received monthly paper reports from health facilities, and submitted monthly digital reports via the District Health Information Software 2 (DHIS2) system, the District Vaccine Data Management Tool (DVD-MT) and the Stock Management Tool (SMT). The DVD-MT and SMT are Excel-based tools, deployed and supported by WHO. The tools were used at the district level for stock management and routine collection of indicator data, using manually entered, monthly aggregate stock data from health facilities. Districts replenished vaccine stock monthly. Replenishment volume was calculated based on the 2012 Tanzania Census, the national targeted vaccination coverage, and per-vaccine wastage estimates. Based on forecasting in the SMT and DVD-MT vaccine was replenished with a target of remaining above 80% of the monthly target population-plus-wastage calculation and not exceeding 120% max value of the target population-plus-wastage calculation and a requirement to maintain a two-week safety stock. If consumption exceeded census estimates, facilities would make a mid-month order to the district to obtain additional supply. Vaccine inventory was physically counted monthly and tracked daily via seven distinct registers (vaccine stock ledgers) and tally sheets.

The Government of Tanzania hoped that a combined EIR-eLMIS at the facility level would help with the following challenges identified in EPI program reviews:

• Incomplete or untimely data, making it hard to respond quickly to issues
• Inaccurate or uncertain denominators for calculating immunization rates
• Difficulty identifying infants who do not start immunization or who drop out (defaulter tracing)
• Lack of unique identifiers for infants
• Poor data visibility into supplies at the facility level to district-level data and stock
• Complex data collection forms and tools
• Insufficient supply chains and logistics management.
• Inadequate data management and use capacity at all levels of the health system

## 2 Intervention, theory of change and hypotheses

### 2.1 Intervention description

Tanzania introduced the combined EIR-eLMIS system, called the Tanzania Immunization Registry (TImR) from June 2016 to January 2018. TImR was then integrated with the Vaccine Information Management System (VIMS)- a district-level digital tool that was simultaneously replacing the SMT and DVD-MT. Roll-out was phased across 26 districts and 924 facilities in the Tanga, Arusha, and Kilimanjaro Regions, serving a population of 6.2 M. TImR is an open-source software developed for use on tablets with online and offline functionality was developed from Open Immunization (OpenIZ), an open source immunization management system. Healthcare workers (HCWs) use TImR to register children receiving vaccinations, document vaccines administered, identify children who are off-schedule, and generate routine aggregate reports of facility.

The Tanzania Ministry of Health, Community Development, Gender, Elderly and Children (MOHCDGEC) through the Immunization and Vaccines Development (IVD) Program led the development of the EIR-LMIS system, with support from PATH and other partners. Tanzania used the Collaborative Requirements Development Methodology (CRDM) and collaborated with health service providers and decision-makers in a User Advisory Group (UAG) to test and develop the package of data quality and use interventions, of which TImR became the core intervention [18], [19]. This approach was intended to bring user perspectives into the design of interventions. Other partners included PORALG, WHO and UNICEF country offices, Mohawk College, CHAI, IntraHealth, JSI, MCSP, GAVI, and BMGF who funded the project.

The five intervention components within the EIR-LMIS system that contribute to stock availability are:

• **Automated deduction in vaccine stock at the facility each time EIR records administered vaccine:** When a health worker records an immunization in the proto-electronic health record, that dose is automatically deducted from the facility’s current stock count, reducing duplicate data entry and saving the health worker time.
• **Low-stock and stock-out notifications on the EIR platform:** When a vaccine commodity’s inventory level exceeds the 120% level or falls below the 80% level, health workers immediately and automatically receive a notification on their tablet. They additionally receive a notification when a stockout occurs.
• **Predictive vaccine requisition using the combination of estimated targeted population and consumption based of children vaccination schedule.** The TimR has functionality of estimating daily, weekly and monthly caseloads of due children that help to inform the actual needs of vaccines during given time period as an additional data to inform amount of vaccines to order and or stock
• **Visualizations of stock levels on the EIR platform, for frontline workers:** Dashboards showing stock levels by commodity let health workers know current stock levels, the level they should be careful to stay above and the level at which they would be overstocked.
• **Automated data exchange with new digital district-level system, the Vaccine Information Management System (VIMS), so that supervisors and frontline workers access same information:** The EIR-eLMIS automatically sends supply chain information to the district-level eLMIS, VIMS. Thus so long as there is network connectivity, district-level staff can access frontline stock information in real-time rather than waiting for monthly reports.
• **Peer networking and targeted supervision to strengthen use of new system:** Health workers in the same area join a WhatsApp peer group in which they share experiences with one another about what worked well or share challenges about what could have been done better and get suggestions from peers. District-level government staff members receive in-depth training on the EIR-eLMIS, then advise health workers if and as challenges arise.

Additional components of the combined EIR-LMIS and other data use interventions included unique identification of both children and commodities using barcodes, clinical dashboards, and an electronic immunization record with automated patient tracking functionality and training guides for data use.

#### 2.1.1 Theory of change and hypotheses

**Fig. 1:**
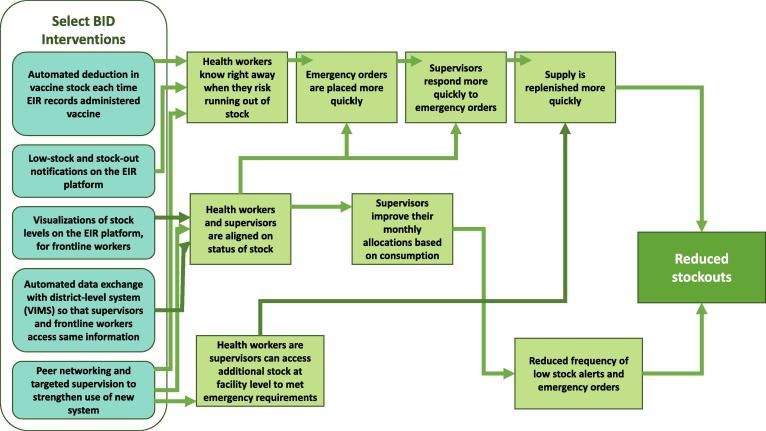
Theory of Change describing how BID interventions lead to improved vaccine availability.

Our intervention theory of change illustrates that this combined package of interventions, and the inclusion of stock notifications, should help improve end-to-end visibility and thereby change both how frontline workers place orders and how supervisors allocate stock across facilities, ultimately resulting in fewer stock-outs over time (see Fig. 1).

Following the deployment of the combined EIR-eLMIS, health workers should know right away when they are at risk of running out of stock: Notifications are intended to reach health workers the moment the system detects that stock has fallen below the desired minimum level. Rather than wait for a physical count or manual calculations, a health worker can use the information they receive from a notification to place an emergency order immediately. Because supervisors can access the same data as the health worker, both align quickly on issues and responsiveness increases. If the needed stock is not available at district level, or the facility is far away, the WhatsApp peer network allows both the health worker and the supervisors to reach out to other facilities that might have overstock and could more quickly and easily replenish the facility in need. The increased responsiveness and increased pool of potential replenishment stock would then result in much faster replenishment response times, as measured from when a facility first falls below their minimum threshold to when they receive the needed stock.

The theory of change suggests that these changes could collectively contribute to improved vaccine availability and reductions in stock-outs.

This study tests the overarching hypothesis that adoption of a linked EIR-eLMIS system will reduce the frequency of stock-outs and thus improve vaccine availability. We also hypothesize that we will see the effect size of the intervention will increase over time. Finally, we hypothesize the overall stockout rates will be lower for vaccines that are actively monitored by the global community and tied to financial incentives—specifically DTP-containing vaccines (DTPcV).

## 3 Methods: Measurement, data, and analytic approach

### 3.1 Study setting

The study was conducted in the Northern Zone of Tanzania, located in the northeast of the country, and included the Arusha Region, Kilimanjaro Region, and Tanga Region. The three regions collectively have a population of 6.2 million and cover a land mass of over 77,500 square kilometres [20]. Some facilities are over one day’s travel away from the district office in remote villages bordering national parks and may see no more than 8 children in a single month. Other facilities are in international, cosmopolitan cities like Arusha and see more than 200 children each day. In total, the three regions are supported by 924 health facilities that provides immunization services.

### 3.2 Study design, data sources and measurement

We used two approaches to estimate the impact of the use of an integrated EIR-eLMIS system on the frequency of vaccine stock-outs. We use a pre/post two-way Analysis of Variance (ANOVA) to test differences in the number of stock-outs before and after intervention roll-out. We used time-series regression modelling to estimate the contribution of time since intervention roll-out on the odds of stock-out.

All data were extracted from two routine datasets from Tanzania’s immunization program: the DVD-MT and TImR. The DVD-MT captures whether a stock-out was observed for a vaccine on the last day of each month. TImR has much more granular data, capturing daily stock balances for each vaccine at the facility level. In order to use both data sources to estimate changes over time for certain analyses presented here, we applied a conservative version of the DVD-MT definition of stock-out to the TImR data (see Table 1). We defined the primary dependent variable (stock-out rate) as a binary variable equalling one (1) if a vaccine’s stock balance ever reached 0 in a facility-month in either DVD-MT or TImR, excluding known data errors in which a true stockout was unlikely—namely a zero-stock event immediately followed by a deduction resulting in a negative stock balance. In analyses using only TImR data (e.g. the interrupted time series) we defined stockouts as occurring in the vaccine-facility-week unit of observation.

**Table 1:** Indicators used for the analysis.

*Indicator*	*Definition*
*Dependent variable*	
Stockout	The existence of any zero-stock event in one or multiple vaccination session in a vaccine-facility-month.

*Independent variables*	
Intervention pre/post	Each observation is tagged as occurring either pre- or post-intervention roll-out.
Type of vaccine	Each observation involves a single vaccine commodity- (e.g. BCG vaccine)- known as a ‘Material’- thus vaccine-specific analyses and comparisons are possible.
Tenure using digital system	Each post-intervention stock event is tagged not only with the date of the transaction, but also the # immunization-days passed between intervention roll-out and that specific transaction. Thus analyses looking at improvements over time since roll-out are possible.

Independent variables included a dummy variable for the intervention period (pre/post), the type of vaccine, and tenure using the TImR system. We used project documents to specify the roll-out month and considered the roll-out month as tenure = 0. We coded the first month of roll-out as pre but all subsequent months as post.

The DVD-MT and TImR datasets used for analysis include 924 of the facilities that received the BID intervention and were further subset based on completeness. The DVD-MT dataset begins in April 2016 in the Arusha Region and January 2017 in the Kilimanjaro and Tanga Regions; for 33 pilot ‘paperless’ facilities in the Tanga Region, the DVD-MT data end upon introduction of TImR. For all remaining facilities, the dataset continues through May 31st, 2018. The TImR dataset begins in July 2017 in the Tanga Region, November 2017 in the Arusha Region, and December 2017 in the Kilimanjaro Region. There were no national shortages of any materials- or vaccine commodities—in the time frame of this study.

All data was released for analysis with permission from the Government of Tanzania, as well as with IRB approval from PATH. The analysis team received anonymized and/or aggregated digital and paper data from the routine program.

### 3.3 Data management and selection criteria

Our analytic approach began with identifying a sample set of facilities that (1) had complete data, and (2) demonstrated adoption of the reporting system by conducting transactions at least once per month both pre- and post-intervention. We assessed completeness of data fields in DVD-MT across all 924 facilities * 6 materials (vaccine commodity types) = 5544 facility-material combinations. First, facilities that were not in the register nor in either system—for example because they do not provide immunization services—were excluded (*n =* 35 facilities, or 210 facility-material combinations). In the pre-intervention data, more than half of the facility-material combinations had missing data for at least one of the months and were thus excluded (*n* = 3083). Next, we excluded facility-materials combinations that had missing data for at least one of the months post-introduction in the digital system (*n* = 1996), leaving 465 facility-material combinations across 144 facilities to evaluate in the pre-post ANOVA.

For facilities with complete data, we then analyzed stock transactions to exclude ‘false stock-outs’. False stock-outs occur when a health worker accidentally enters a historic vaccination as a current vaccination; thereby incorrectly triggering a deduction from vaccine stock. Anytime a zero or negative-balance transaction was followed by a subsequent deduction, it was excluded from ‘stock-out’ classification. 43 facilities had at least one false stock-out, but negative stock-outs never exceeded 3% of transactions for any facility at any point in time.

Table 2 describes the characteristics of facilities included in the analysis compared to the broader set of facilities. While each antigen had complete data from roughly 10% of all facilities, only 23 facilities total (<5% of facilities) had complete data for all antigens, with over half of facilities in the sample having three or fewer antigens with complete data. Kilimanjaro Region did not have a single facility with complete data and so no facilities from that region were included in the sample. The distribution of facility levels is similar across the sample and full facility set, with a slight overrepresentation of lower-level facilities (e.g. dispensaries) in the sample set. In terms of facility characteristics, double-sided t-tests showed that the sample facilities in Arusha had a higher average of immunization days compared to all facilities (tstat = −2.38; *p* = 0.019), significantly fewer private sector facilities (tstat = 7.72; *p* < 0.01) and were further on average from the district health office (tstat = −4.14, *p* < 0.01).

**Table 2:** Comparison of facilities used in ANOVA sample with data available on all facilities.

	Sample facilities	All facilities	T	[95% CI]	p
*Region*					
Tanga (%)	0.64*** (n = 144)	**0.37** (n = 889)	36.99	[-0.361, −0.184]	0.000***
Arusha (%)	**0.36** (n = 144)	**0.31** (n = 889)	1.12	[-0.137, 0.040]	0.29
Kilimanjaro (%)	0*** (n = 144)	**0.32** (n = 889)	62.16	[0.285, 0.355]	0.000***

TANGA					
*Levels*					
Dispensary (%)	**0.880** (n = 92)	**0.865** (n = 326)	0.045	[-0.098, 0.068]	0.833
Health Center (%)	**0.109** (n = 92)	**0.110** (n = 326)	0.000	[-0.072, 0.076]	1
Hospital (%)	**0.011** (n = 92)	**0.025** (n = 326)	0.153	[-0.020, 0.048]	0.696

*Facility Characteristics*					
Number HCWs (mean)	**2.33** (n = 73)	**2.46** (n = 267)	0.91	[-0.158, 0.429]	0.362
DVDMT doses/month (mean)	**300.72** (n = 92)	**323.29** (n = 314)	0.76	[-36.248, 81.375]	0.450
Distance to DHO (mean)	**26.49** (n = 91)	**23.49** (n = 318)	−1.94	[-6.051, 0.058]	0.054
Private ownership (%)	**0.077** (n = 91)	**0.123** (n = 318)	1.06	[-0.027, 0.118]	0.304

ARUSHA					
*Levels*					
Dispensary (%)	**0.81** (n = 47)	**0.78** (n = 188)	0.04	[-0.152, 0.808]	0.845
Health Center (%)	**0.15** (n = 47)	**0.17** (n = 188)	0.00	[-0.104, 0.134]	0.946
Hospital (%)	**0.04** (n = 47)	**0.05** (n = 188)	0.00	[-0.058, 0.074]	1

*Facility Characteristics*					
Number HCWs (mean)	**2.23** (n = 47)	**2.20** (n = 181)	−0.242	[-0.324, 0.254]	0.809
DVDMT doses/month (mean)	**333.00** (n = 52)	**341.34** (n = 221)	0.120	[-130.07, 146.76]	0.905
Distance to DHO (mean)	55.03*** (n = 49)	**39.76** (n = 265)	−4.144	[–22.59, −7.95]	0.000***
Private ownership (%)	0.12*** (n = 49)	**0.33** (n = 271)	7.716	[0.090, 0.329]	0.005***

*Additional characteristics only captured in Arusha Region*					
Immunization days per week (mean)	4.66** (n = 47)	**4.19** (n = 176)	−2.38	[-0.866, −0.078]	0.019**
Has internet (%)	**0.81** (n = 47)	**0.89** (n = 188)	1.49	[-0.055, 0.214]	0.222
High volume (%)	**0.70** (n = 46)	**0.66** (n = 190)	0.098	[-0.200, 0.125]	0.754

*p < 0.1.

**p < 0.05.

***p < 0.01.

### 3.4 Statistical analysis

#### 3.4.1 Before-after comparison

For facilities with complete data and minimal known data errors, we compared vaccine availability from two time periods: average availability for the six months prior to intervention using DVD-MT data, and average availability for the six months post intervention using TImR data. We use a two-way Analysis of Variance (ANOVA) with the independent variables being time period and type of vaccine.

#### 3.4.2 Mixed-effects logistic regression models

Acknowledging the limitations of comparing two different data sources over time, we performed a secondary analyses only using TImR data post-introduction. We estimated the odds of a facility-week-antigen stock-out in a mixed-effects logistic regression model to test the hypothesis that the odds of a stock-out would decrease over time corresponding to the amount of time a facility had been using TIMR. We subset our analysis to facilities in Arusha and Tanga Regions only.

The logistic regression model included a mixed-effect for facility. We included covariates in the final model if they were statistically significant in univariate mixed-effects models. The final model included the continuous covariate for weeks of tenure with TIMR, a covariate for antigen, and an interaction term for weeks of tenure and antigen.

ANOVA analyses were performed in Microsoft Excel (Redmond, WA) and regression analyses in Stata Version 12 (College Station, TX).

## 4 Findings

### 4.1 Descriptive trends in availability

**Fig. 2:**
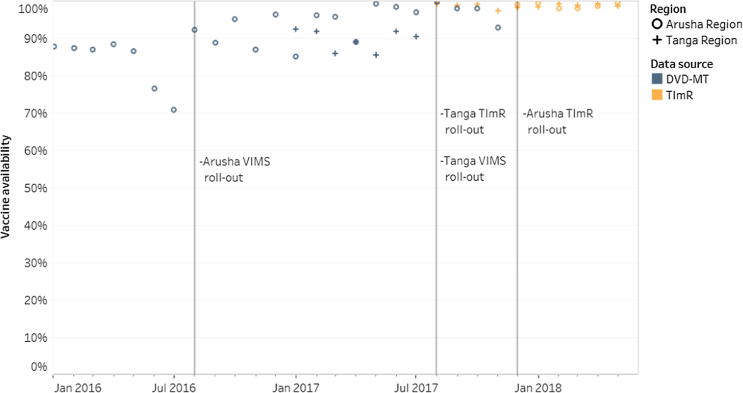
Percent of vaccine available across all facility-antigens in sample.

Fig. 2 describes the overall trend in vaccine availability, over time, for the included facilities in Tanga and Arusha Regions. Timestamps show three key intervention points: the introduction of VIMS, the introduction of TImR in Tanga Region and the introduction of TImR in Arusha Region. Arusha Region was part of a VIMS pilot. In Tanga Region, VIMS and TImR were introduced at the same time.

### 4.2 Pre-post ANOVA of stockout frequency

Table 3 provides an overview of outcomes from the pre-post ANOVA. Post-introduction, overall stock-outs declined from a monthly average of 7.1% to 2.1% *(p* < 0.01). That is, in the six months prior to the intervention 7.1% of vaccine-facility-month observations experienced any stock-out compared to 2.1% in the six months following the intervention roll-out. Three specific vaccines also had statistically significant declines in monthly stock-out frequency: OPV’s monthly average dropped from 12.5% to 2.1% (*p* < 0.01), MR from 9.4% to 1.0% (*p* < 0.01) and DTP-HepB-HiB from 8.1% to 1.7% (*p* < 0.01). Three antigens did not have a statistically significant difference in stockout frequency pre- and post-intervention. Those were BCG, PCV13 and Rota vaccines.

**Table 3:** Monthly stockout frequency, by antigen, pre- and post-intervention.

Antigen	# facility-antigens in cohort	Six months pre-intervention, DVD-MT			Six months post-intervention, TImR		
		*# stock-outs*	*Average*	*Variance*	*# stock-outs*	*Average*	*Variance*
BCG	66	19	4.8%	0.36	9	2.3%	0.21
OPV***	79	59	12.4%	1.04	10	2.1%	0.14
MR***	69	39	9.4%	1.04	4	1.0%	0.06
PCV13	87	20	3.8%	0.25	14	2.7%	0.28
ROTA	84	22	4.4%	0.24	11	2.2%	0.16
DTPcV***	80	39	8.1%	0.63	8	1.7%	0.14
Total***	465	198	7.1%	0.61	56	2.0%	0.17

*p < 0.1.

**p < 0.05.

***p < 0.01.

### 4.3 Evaluation of the impact of tenure and vaccine type: mixed-effects logistics regression model

Table 4 describes the estimates from the mixed-effects logistic regression on TImR data from Arusha and Tanga Regions. The final model includes covariates for week of tenure with TImR and vaccine. Controlling for antigen, the odds of stockout are 4.1% (95% CI: 3.3 – 4.9) lower for each week of tenure with the system. Controlling for time, the odds of stockout varied by antigen. Compared to DTPcV vaccine, the odds of BCG vaccine being stocked out were 4.31 as high (95% CI: 3.1 – 5.0). The odds of being stocked-out were 29.7% lower for PCV (95% CI: 8.8 – 45.8) and 26.6% (95% CI: 3.4 – 44.1) lower for rotavirus vaccines compared to DTPcV. The odds of stock out were 37.7% lower for MR vaccine than DTPcV (95% CI: 18.1 – 52.6). In other words, BCG was more likely to be stocked out than DTPcV and rotavirus, PCV and MR vaccines were less likely to be stocked out than DTPcV, controlling for time. The variance of the random effect for facility was also statistically significant.

**Table 4:** Coefficients from mixed-effects logistic regression model: odds of stock-out.

Model covariates	Odds ratio	SE	95% CI
Weeks of tenure with the system	0.959***	(0.00419)	0.951–0.967
BCG	3.955***	(0.462)	3.145–4.973
OPV	0.841	(0.118)	0.639–1.107
PCV	0.703***	(0.0932)	0.542–0.912
Rotavirus	0.734**	(0.103)	0.559–0.966
MR	0.623***	(0.0867)	0.474–0.819
Constant	0.000315***	(0.000140)	0.000132–0.000753
Variance of facility random effect	3.879***	(0.237)	3.441–4.372
Observations	68,472		
Number of groups	505		
seEform in parentheses			
*p < 0.01			
**p < 0.05			
***p < 0.01			

## 5 Discussion

The results confirm some hypotheses, while also raising a number of additional questions. The findings from the pre-post ANOVA confirm that after the combined EIR-LMIS with stock notification was introduced, vaccine availability increased for three of the six evaluated vaccines and for overall vaccine availability. The mixed-effects logistics regression model confirm that the longer health workers had with the digital system, the less likely they were to experience stock-outs for PCV13, rotavirus and MR vaccines, compared to DTP-HepB-HiB. This finding suggests that longer tenure with a digital information system may contribute to reduced stock-outs over time once that system is introduced. This would validate many change management and technology adoption theories [21], [22], [23], [24]. It also suggests that digital implementations require a certain amount of time following introduction before demonstrating their effectiveness.

The results partly confirmed our hypothesis that some vaccine commodities would be statistically more likely to be stocked out than others. However, despite being widely tracked and used for global incentives programs, DTP-HepB-HiB was *not* the most likely to be consistently available. It’s true that OPV has additional incentives and pressures given the global Polio eradication agenda, and some of the other vaccines (PCV13, rotavirus) are newer in Tanzania’s vaccination program. Similarly, no discernible pattern emerges in terms of delivery mechanism (oral vs injectable), cost per dose nor the number of doses per vial. One possible explanations of note: BCG may be more likely to be stocked out because it may be more commonly administered in maternity clinics rather than immunization clinics where this intervention was deployed. Immunization clinics rarely see children at birth, when BCG is administered to those born at a health facility.

In screening for data completeness, the pre-post analysis highlighted adoption challenges both with the legacy (DVD-MT) and the combined EIR-LMIS systems. Less than 20% of facilities collecting data were included in the analysis, and the entire region of Kilimanjaro was excluded, due to incomplete data. Our analysis sample in Arusha was slightly skewed towards facilities that were more likely to be privately owned, further from the district health office and with a greater number of immunization days per week. This selection bias limits the generalizability of our findings to other facilities that use the EIR-eLMIS system; as observed in other analyses of these data, system use is associated with facility type (health centers and hospitals being more likely to use the system than dispensaries), number of health workers trained on the system, and varied significantly by district and week since system introduction.

We posit that facilities with incomplete data in the legacy system are more likely to be constrained on a range of facility-readiness dimensions; not being able to estimate the effect of the intervention on low-performing facilities is a potential limitation. During the time of this study, the legacy system remained as the official reporting system, and this could in part explain why the legacy system had 353 facilities submitting complete data while not using the combined EIR-eLMIS system. While the findings of this paper present important conclusions related to supply chain, these findings are constrained to where adoption is successful and data are complete. Additional research and investigation is required to understand why adoption rates were initially low in the legacy system and remained low in the digital system.

Before-after studies and time series analyses can accurately depict changes over time, but there is some risk that attribution for those observations is due to external events occurring at the same time. External factors impacting datasets—such as the loss of key health personnel, the recruitment of highly motivated new staff, or even unanticipated cointerventions to reduce stockouts—may show up in data and may distort month-to-month findings. While no national-level stockouts occurred during the study period, the sub-national fluctuations in the availability of specific vaccines- particularly at the regional level, could be one explanation for the different impacts across antigens. Furthermore, completeness criteria may have excluded low-performing facilities. This exclusion may have resulted in an under-estimation of impact if facilities with no ‘pre’ data showed marked improvement, but more likely this exclusion resulted in a sample of higher-performing facilities overall and thus the results are likely more relevant for high-performing facilities rather than low-performing ones. The ANOVA was conducted without replication; where facilities contributed data to more than one antigen, it’s likely they would have consistent results across antigens and the model did not account for this. While the time series regression analysis helps describe the timeline to stability, it does not answer the question of whether having digital solutions leads to higher impact than not having a digital solution. It’s also important to note that attribution cannot easily be made to any one part of the EIR-LMIS intervention package, as all interventions were implemented together. However, this study does provide an opportunity to add to a growing body of literature on the impact of integrated, combined digital interventions. Finally, all data used in this study was collected via a routine health management information system. Compared to datasets from clinical trials or surveys, this dataset has lower levels of completeness and lower levels of quality, though the methods described should have largely mitigated these risks. Multiple, imperfect datasets allowed for some degree of triangulation, but added quality issues in the sample set and reduced the overall sample size due to completeness challenges. On the other hand, we demonstrated the utility of using routine health data as part of timely, real-world evaluation [25]. In particular, the automated capture of new process indicators and granular data can enable researchers to examine new types of questions, and more thoroughly answer how an intervention works, not just whether it works.

## 6 Conclusion

This research provides compelling evidence that vaccine availability patterns are heterogeneous across commodities, and that tenure with an integrated EIR-eLMIS system decreases the odds of a stockout. It additionally provides promising evidence suggesting that the combined EIR-LMIS can improve vaccine availability compared to pre-introduction availability rates. Further investigation into how health workers are currently using the legacy and new system in facilities with both, an in-depth evaluation of the assumptions along the proposed theory of change, and triangulation across national stock data, cold chain equipment data, survey data and home-based records would help confirm the veracity of various data sources within routine systems. It would additionally deepen global understanding of both the value of digital supply chain interventions, as well as the level of investment and change management support required to achieve a successful deployment. This research illustrates how digital interventions can act as catalysts to strengthen components of programs of known efficacy – indirectly impacting priority maternal and child health outcomes [26].

Beyond vaccine availability, additional research is needed to understand the impact of the combined EIR-LMIS on vaccine wastage, how open vial wastage data can contribute to triangulations for data quality assessments, vaccine replenishment response times and facility- or individual- level drivers of strong vaccine supply chain performance (e.g. facility size, performance, individual tenure within the broader health system, …etc.). These research areas, as well as a qualitative root cause analysis of results, would strengthen interpretation of these results and provide stronger evidence to validate or challenge the proposed theory of change.

## Declaration of Competing Interest

The authors declare that they have no known competing financial interests or personal relationships that could have appeared to influence the work reported in this paper.

## Footnotes

1 These antigens are: Tuberculosis, Poliovirus, Diphtheria, Tetanus, Pertussis, Hepatitis B, Haemophilus influenzae type B, Rotavirus, Pneumococcus, Measles, Rubella and Human papillomavirus.
